# Supplementary material for: Evaluating the implementation of an early supported discharge (ESD) program for stroke survivors: A mixed methods longitudinal case study
Source: PLoS One. 2020 Jun 24;15(6):e0235055. doi: 10.1371/journal.pone.0235055 (PMC7313954; doi:10.1371/journal.pone.0235055)
Supplement: S1 Data — (DOCX) [file pone.0235055.s001.docx]

| **Domain** | **Construct** | **Referrer Survey** | **Clinician Survey** | **Clinician Focus Group** | **Question** |
| --- | --- | --- | --- | --- | --- |
| Intervention Characteristics | Intervention Source |  | X |  | Scale = 0 very poor to 4 excellent   - Your knowledge of who developed ESD - Your understanding of why ESD is being used in your setting |
|  | Evidence Strength & Quality |  | X |  | Scale = 0 very poor to 4 excellent   - The quality of evidence available in regards to whether ESD works with your patient population |
|  | Relative Advantage |  | X |  | Scale = 0 very poor to 4 excellent   - How ESD compares to other similar existing programs in your setting - How ESD compares to other alternatives you may have considered or that you know about |
|  | Adaptability |  |  |  | Scale = 0 very poor to 4 excellent   - The degree to which you can adapt ESD to work effectively in your setting - The amount of autonomy your have to adapt ESD to work effectively in your setting |
|  | Complexity |  | X |  | Scale = 0 not complex to 4 extremely complex   - The overall complexity of ESD (i.e. the number of steps / people / resources involved) - The duration of ESD - The scope of ESD - The intricacy of ESD - The number of steps involved in ESD - The degree of difference to previous practice |
|  | Design Quality & Packaging |  | X |  | Scale = 0 very poor to 4 excellent   - Your perception of the quality of the supporting materials and resources for ESD - The degree to which supporting materials and resources enable ESD |
|  | Cost |  | X |  | Scale = 0 no impact to 4 severe impact   - The impact of costs on the implementation of ESD |
| Outer Setting | Patient Needs & Resources |  |  | X | To what extent were the needs and preferences of the individuals served by your organization considered when deciding to implement the intervention? |
|  | Cosmopolitanism |  |  | X | Can you tell me what you know about any other organizations that have implemented the intervention or other similar programs? |
|  | Peer Pressure |  |  | X | To what extent would implementing the intervention provide an advantage for your organization compared to other organizations in your area? |
|  | External Policy & Incentives |  |  | X | What kind of local, state, or national performance measures, policies, regulations, or guidelines influenced the decision to implement the intervention?  What kind of financial or other incentives influenced the decision to implement the intervention? |
| Inner Setting | Structural Characteristics |  |  | X | How did the infrastructure of your organization (social architecture, age, maturity, size, or physical layout) affect the implementation of the intervention?  What kinds of infrastructure changes will be needed to accommodate the intervention? |
|  | Networks & Communications |  |  | X | Who do you ask if you have questions about the intervention or its implementation? |
|  | Culture |  |  | X | How well did the intervention fit with your values, norms and goals within the organization? |
|  | Implementation Climate | X |  | X | Scale = 0 no impact to 4 severe impact   - Impact on ESD inpatient workload   Is there a strong need for this intervention?  How well did the intervention fit with existing work processes and practices in your setting? |
|  | Readiness for Implementation | X | X | X | Scale = 0 very poor to 4 excellent   - Education or information you have received about ESD   Scale = 0 I have knowledge of key aspects of ESD, but don’t offer it regularly to 4 I recognise the benefits of ESD, have integrated it into routines and promote its use to others   - Readiness to implement   What kinds of information and materials about the intervention have already been made available to you?  Did you have sufficient resources to implement and administer the intervention? |
| Characteristics of Individuals | Knowledge & Beliefs about Intervention | X | X |  | Scale = 0 very poor to 4 excellent   - Your overall knowledge of ESD - Your overall perception of ESD - Your understanding of how to identify patients who might be suitable for ESD   Scale = 0 very poor to 4 excellent   - Your overall knowledge of ESD - Your overall perception of ESD |
|  | Self-Efficacy |  | X |  | Scale = 0 very poor to 4 excellent   - Your confidence in your ability to successfully implement ESD - Your colleagues confidence in their ability to successfully implement ESD |
|  | Individual Stage of Change |  |  | X | What is the general level of receptivity in your organization to implementing the intervention? |
|  | Individual Identification with Organisation |  | X |  | Scale = 0 very poor to 4 excellent   - The degree to which your organisational goals align with using ESD |
| Process | Engaging | X |  | X | Scale = 0 very poor to 4 excellent   - Ease of communication with ESD coordinator / project lead   What level of endorsement or support have you seen or heard from leaders? |
|  | Executing | X |  |  | Scale = 0 very poor to 4 excellent   - Ease of the referral process - Ease of transfer between hospital and ESD |
|  | Reflecting & Evaluating |  |  |  | Scale = 0 very poor to 4 excellent   - Satisfaction with the referral process - Satisfaction with transfers between hospital and ESD - Satisfaction with communication with ESD coordinator/project lead   Scale = 0 very poor to 4 excellent   - ESD effectiveness in your area - Your satisfaction in providing ESD to patients   How well do you think the intervention met the needs of the individuals served by your organization? |
