# Supplementary material for: Evaluating the implementation of an early supported discharge (ESD) program for stroke survivors: A mixed methods longitudinal case study
Source: PLoS One. 2020 Jun 24;15(6):e0235055. doi: 10.1371/journal.pone.0235055 (PMC7313954; doi:10.1371/journal.pone.0235055)
Supplement: S2 Data — (DOCX) [file pone.0235055.s002.docx]

**Referrers Survey**

**Please circle**

Location: XXXX Hospital Other______________________

XXXX Hospital

Discipline: Physiotherapist Occupational Therapist

Allied Health Assistance Dietician

Social Work Speech Pathology

Psychologist

Other: (Please state)

Please answer the following questions in relation to your perceptions of the Early Supported Discharge (ESD) program at this time.

| **Please rate the following** | | Very poor | Fair | Average | Good | Excellent |
| --- | --- | --- | --- | --- | --- | --- |
| 1 | Your overall knowledge of ESD | **0** | **1** | **2** | **3** | **4** |
| 2 | Your overall perception of ESD | **0** | **1** | **2** | **3** | **4** |
| 3 | Education or information you have received about ESD at Western Health | **0** | **1** | **2** | **3** | **4** |
| 4 | Your understanding of how to identify patients who might be suitable for ESD | **0** | **1** | **2** | **3** | **4** |
| 5 | Ease of the referral process | **0** | **1** | **2** | **3** | **4** |
| 6 | Satisfaction with the referral process | **0** | **1** | **2** | **3** | **4** |
| 7 | Ease of communication with ESD coordinator / project lead | **0** | **1** | **2** | **3** | **4** |
| 8 | Satisfaction with communication with ESD coordinator / project lead | **0** | **1** | **2** | **3** | **4** |
| 9 | Ease of transfer between hospital and ESD | **0** | **1** | **2** | **3** | **4** |
| 10 | Satisfaction with transfer between hospital and ESD | **0** | **1** | **2** | **3** | **4** |
| 11 | Impact of ESD on inpatient staff workload | **0** | **1** | **2** | **3** | **4** |

Are there any other aspects of ESD you would like to comment on? Positives? Negatives?

Unexpected experiences? You may write on the back of this sheet if you have further comments.

Thank you very much for your time

**Delivering Clinician Survey**

**Please circle**

Location: Community Rehabilitation at XXXX Hospital

Community Rehabilitation at XXXX Hospital

Discipline: Physiotherapist Occupational Therapist

Allied Health Assistance Dietician

Social Work Speech Pathology

Psychologist

Other: (Please state)

Please answer the following questions in relation to your perceptions of the Early Supported Discharge (ESD) program over the past fortnight.

| **Please rate the following** | | Very poor | Fair | Average | Good | Excellent |
| --- | --- | --- | --- | --- | --- | --- |
| 1 | Your overall knowledge of ESD | **0** | **1** | **2** | **3** | **4** |
| 2 | ESD effectiveness in your setting | **0** | **1** | **2** | **3** | **4** |
| 3 | Your overall perception of ESD | **0** | **1** | **2** | **3** | **4** |
| 4 | Your confidence in your ability to successfully implement ESD | **0** | **1** | **2** | **3** | **4** |
| 5 | Your colleagues confidence in their ability to successfully implement ESD | **0** | **1** | **2** | **3** | **4** |
| 6 | The degree to which your organizational goals align with using ESD | **0** | **1** | **2** | **3** | **4** |
| 7 | Your satisfaction in providing ESD to your patients | **0** | **1** | **2** | **3** | **4** |
| 8 | Your knowledge of who developed ESD | **0** | **1** | **2** | **3** | **4** |
| 9 | Your understanding of why ESD is being used in your setting | **0** | **1** | **2** | **3** | **4** |
| 10 | The quality of evidence available in regards to whether ESD works with your patient population | **0** | **1** | **2** | **3** | **4** |
| 11 | The quality of evidence available in regards to whether ESD works with your patient population | **0** | **1** | **2** | **3** | **4** |
| 12 | How ESD compares with other similar existing programs in your setting | **0** | **1** | **2** | **3** | **4** |
| 13 | How ESD compares to other alternatives you may have considered or that you know about | **0** | **1** | **2** | **3** | **4** |
| 14 | The degree to which you can adapt ESD to work effectively in your setting | **0** | **1** | **2** | **3** | **4** |
| 15 | The amount of autonomy you have to adapt ESD to work effectively in your setting | **0** | **1** | **2** | **3** | **4** |
| 16 | Your perception of the quality of the supporting materials and resources for ESD | **0** | **1** | **2** | **3** | **4** |
| 17 | The degree to which supporting materials and resources enable ESD | **0** | **1** | **2** | **3** | **4** |

| **Please rate the following** | | Not complex | Slightly complex | Somewhat complex | Very complex | Extremely complex |
| --- | --- | --- | --- | --- | --- | --- |
| 18 | The overall complexity of ESD (i.e. the number of steps / people / resources involved) | **0** | **1** | **2** | **3** | **4** |
| 19 | The duration of ESD | **0** | **1** | **2** | **3** | **4** |
| 20 | The scope of ESD | **0** | **1** | **2** | **3** | **4** |
| 21 | The intricacy of ESD | **0** | **1** | **2** | **3** | **4** |
| 22 | The number of steps involved in ESD | **0** | **1** | **2** | **3** | **4** |
| 23 | The degree of difference to previous practice | **0** | **1** | **2** | **3** | **4** |

| **Please rate the following** | | No Impact | Slight Impact | Moderate Impact | Considerable Impact | Severe Impact |
| --- | --- | --- | --- | --- | --- | --- |
| 18 | The impact of costs on the implementation of ESD | **0** | **1** | **2** | **3** | **4** |

Which of the following reflects how prepared you are to use ESD

| 1 | I have knowledge of key aspects of ESD, but don’t offer it regularly | **0** |
| --- | --- | --- |
| 2 | I like ESD, discuss it with others, buy into it and have a positive view. I offer ESD regularly | **1** |
| 3 | I’ve sought additional information about ESD and offer ESD regularly | **2** |
| 4 | I have acquired additional information, use ESD regularly, and will continued to use it for the foreseeable future | **3** |
| 5 | I recognize the benefits of ESD, have integrated it into routines, and promote its use to others | **4** |

Thank you for your time
