# Supplementary material for: Evaluating the implementation of an early supported discharge (ESD) program for stroke survivors: A mixed methods longitudinal case study
Source: PLoS One. 2020 Jun 24;15(6):e0235055. doi: 10.1371/journal.pone.0235055 (PMC7313954; doi:10.1371/journal.pone.0235055)
Supplement: Attachment — Submitted filename: Plosone_ESD_Oct19.docx [file pone.0235055.s003.docx]

Evaluating the implementation of an Early Supported Discharge (ESD) program for stroke survivors: A mixed methods longitudinal case study

This paper reports findings from a study aimed at describing staff perceptions of the implementation of an ESD model of care for stroke survivors. The setting was a public health service in a major city in Australia.

General comments

I would like to thank the authors for reporting the findings from this study – there has been a lack of implementation of Early Supported Discharge services in Australia and so this will be of interest to the stroke community. However, I do think the paper needs considerably more work to get it to the standard required for publication and to ensure a reader would be able to interpret the findings correctly.

Overall this paper is very long and as a consequence key messages were lost. Whilst use of the Consolidated Framework of Implementation Research (CFIR) provided a sound theoretical base for the study, over-use of CFIR domain terms (without much explanation) made the paper very difficult to read. The results section would benefit from being made much more conscise, with more explanation and emphasis of the link between the domains featured in the survey and themes (and quotes) derived from the interviews and focus groups.

The research team’s use of mixed methods was good, however rationale or explanation for the phasing of the study was lacking. Description of changes in survey scores I also felt was misleading – e.g. ‘increased markedly’ when changes were not significant.

Use of the term ‘ESD trial’ also confused me. I initially thought the study was investigating ESD in real world settings – some of the findings suggested the focus was more related to implementation ESD as a trial-based intervention. This has important implications for how findings are interpreted and used in future research or in practice.

The discussion also lacked a clear summary of key findings, with again a strong focus on the CFIR, without clear articulation of how the study added to current literature relating to the implementation of rehabilitation services or ESD for stroke survivors.

Details

Background – I would question some of the referencing – referencing seems to be based on comments made in a paper, rather than using the reference for the original source e.g. [3] is a review not a global consensus paper.

Materials and Methods – Would benefit from being clear if the intention is to investigate implementation of ESD in a trial, or in routine practice.

Interviews and/or focus groups – Need to provide rationale for use of each and with what participants

Phases 1 and 2 in the table are not explained in the text

Statistical methods section is weak and requires more detail and justification – independent groups or repeated measures?

Results

Table 1 – Did participants of the baseline survey participate in survey completion at later time points (relates to statistical comparisons)?

Tables 2,3,4,5 – These would benefit from being collapsed into more succinct tables with more explanation about domains. Scoring (out of what?) and statistical analyses also needs to be explained in a brief footnote. Methods says two bespoke surveys were used for each participant group – yet these groups were compared in the tables and text. How was that possible?

Discussion

I commend the research team for focusing on the CFIR and for a detailed discussion about aspects of implementation. However, I couldn’t follow some of the points I would also question the conclusion.
