# Supplementary material for: Evaluating the implementation of an early supported discharge (ESD) program for stroke survivors: A mixed methods longitudinal case study
Source: PLoS One. 2020 Jun 24;15(6):e0235055. doi: 10.1371/journal.pone.0235055 (PMC7313954; doi:10.1371/journal.pone.0235055)
Supplement: Attachment — Submitted filename: PloS One Response to_Reviewers.docx [file pone.0235055.s004.docx]

Dear Academic Editor Tan,

**RE:	PONE-D-19-26093 - Evaluating the implementation of an Early Supported Discharge (ESD) program for stroke survivors: A mixed methods longitudinal case study**

Thank you for your email dated October 31^st^, 2019 regarding this manuscript. We have endeavoured to answer all of the reviewer’s queries and recommendations to the best of our ability, and please find attached a point-by-point response to all of their comments.

In regards to data sharing, there are ethical restrictions associated with the de-identified data set associated with the conduct of this study as a case study. Even with de-identification, participants may be identifiable (particularly those who are members of the smaller allied health professions). The research team are in principle willing to share the data on request, once it has been reviewed by the approving Ethics Committee to ensure there is no risk of inadvertent identification. The contact details for this Committee are: Office of Research, Western Health, Level 3 Western Centre for Health, Research & Education (CHRE), Sunshine Hospital, Furlong Road, St Albans Victoria 3021, Australia.

We look forward to continuing to participate in the peer review process with PLOS One in the coming weeks.

Kind regards,

Dr. Danielle Hitch

Corresponding Author

| **Reviewer 1** |  |
| --- | --- |
| Overall this paper is very long and as a consequence key messages were lost. Whilst use of the Consolidated Framework of Implementation Research (CFIR) provided a sound theoretical base for the study, over-use of CFIR domain terms (without much explanation) made the paper very difficult to read. The results section would benefit from being made much more concise, with more explanation and emphasis of the link between the domains featured in the survey and themes (and quotes) derived from the interviews and focus groups. | A brief description of each domain and construct has now been added into the Methods section to provide easier access to these terms for readers.  The results section has now been condensed, and quantitative / qualitative findings amalgamated into the tables. By placing quotes alongside the quantitative findings, and streamlining the accompanying elaboration, we hope the links between the two forms of data have been made clearer. |
| The research team’s use of mixed methods was good, however rationale or explanation for the phasing of the study was lacking. Description of changes in survey scores I also felt was misleading – e.g. ‘increased markedly’ when changes were not significant. | A paragraph within the methods has now been redeveloped to provide an additional rationale for the use of phasing.  The descriptions of change have now all been revised to remove potentially misleading qualifiers. |
| Use of the term ‘ESD trial’ also confused me. I initially thought the study was investigating ESD in real world settings – some of the findings suggested the focus was more related to implementation ESD as a trial-based intervention. This has important implications for how findings are interpreted and used in future research or in practice. | The implementation of ESD has been framed as a trial throughout the manuscript, and this has been further emphasised in the revised paragraph regarding the model of ESD adopted. |
| The discussion also lacked a clear summary of key findings, with again a strong focus on the CFIR, without clear articulation of how the study added to current literature relating to the implementation of rehabilitation services or ESD for stroke survivors. | The discussion now begins with a paragraph explicitly linking the findings to previous findings regarding the implementation of ESD for stroke survivors. |
| Background – I would question some of the referencing – referencing seems to be based on comments made in a paper, rather than using the reference for the original source e.g. [3] is a review not a global consensus paper. | This appears to have been an error in the referencing, which has now been corrected. All other references have been checked and appear to be correct. |
| Materials and Methods – Would benefit from being clear if the intention is to investigate implementation of ESD in a trial, or in routine practice. | Additional references have been added to the methodology to clearly highlight this evaluation was undertaken in the context of a trial. |
| Interviews and/or focus groups – Need to provide rationale for use of each and with what participants | An additional sentence has been added to explain these formats were offered to enable participant choice, and reduce any potential burden (i.e. participants may not have been able to physically attend a focus group, but were able to take part in a phone interview). |
| Phases 1 and 2 in the table are not explained in the text | An explanation of the phases are now provided in the text of the methodology. |
| Statistical methods section is weak and requires more detail and justification – independent groups or repeated measures? | Additional sentence has been added to specify the groups were considered independently, which is also consolidated by the additional information about the phases. |
| Results |  |
| Table 1 – Did participants of the baseline survey participate in survey completion at later time points (relates to statistical comparisons)? | Please see above |
| Tables 2,3,4,5 – These would benefit from being collapsed into more succinct tables with more explanation about domains. Scoring (out of what?) and statistical analyses also needs to be explained in a brief footnote. Methods says two bespoke surveys were used for each participant group – yet these groups were compared in the tables and text. How was that possible? | The scoring out of X has now been added to the table. Additional statistical information has also been added to the notes below each table. Please see previous comments regarding additional information provided about domains and constructs. While the surveys were bespoke for each group, the same questions were asked of both groups for the variables where they are compared (albeit with slightly different wording). All questions and their alignment to the CFIR (and each group) is available in Supplementary Material S1, and an additional question has been added to the manuscript to clarify this further. |
| **Reviewer 2** |  |
| Recommend cutting the word count by half or more. | It was not possible to cut the words in this document by such as large amount, without losing some essential information. However, we have been able to achieve a 26% decrease in the length of the article (originally 9964 words, now 7670 words) with significant editing. |
| Introduction The ESD program that has been implemented is never described. These programs vary a tremendous amount. There is no standard program at this time. Without an explanation of the program the reader has no context for understanding the results. | An additional paragraph has been added with a description of the ESD program implemented, with reference to general descriptions of the key features of this model of care. |
| Material and methods: Line 89 …explore the complexities-unclear Do you mean the staff and referrer perspective? There is an overuse of the word “complexities” in the manuscript which actually makes understanding the writing more complex. Simply state what you mean. | All references to this term have been reviewed, and alternative terms used in all but one case. Other remaining uses of the term ‘complexity’ are in specific relation to that construct in the CFIR. |
| Line 95 …”a partially embedded implementation research partnership” –unclear. | Additional information has been provided about this term to explain it in further detail. |
| Line 113 cite your other publication | This citation has now been added in |
| Participants Line 118-9 define who these people are. For instance, “staff” in this section is all who are involved in planning, implementing and delivery. However, in the tables staff only refers to “delivering staff”. It is very difficult to understand who was involved in what aspects of the study throughout this manuscript. Further clarify exactly who was took which surveys and who was involved in the focus groups. Was any other data collected regarding demographics: Age, race, years in the profession, years employed at that hospital? Were they all stroke experts? | Additional information has been added throughout the methodology to clarify who took part in the study, and how their data was collected. None of the demographics you mention were collected, due to ethical concerns related to inadvertent identification given the case study method. All of the clinicians included in this study worked with stroke survivors, but their status as stroke experts was not measured. |
| Data Collection This entire section is unclear. The use of CFIR is a positive aspect of this study, but the explanation of the framework and how it is used is poorly executed. The reviewer had to look up the framework and read someone else’s explanation for clarification. It seems like both one-on-one interviews and focus groups were conducted. How many occurred and what was the make-up of each? Was it semi-structured? What method was used to collect the data and what questions were asked or, at least, what constructs were covered? It would be helpful to include the surveys as a supplement. | A brief description of each domain and construct has now been added into the Methods section to provide easier access to these terms for readers. Further details regarding the number of interviews / focus groups and their make up have been added to the manuscript at a level which preserves the privacy of the attendees. Additional detail added to confirm interviews were semi-structured. The method used to collect this data (i.e. face to face and telephone interviews) is referred to in the manuscript, and all questions are provided in Supplementary Material S1. The surveys are now included as Supplementary Material S2. |
| Outcome Measures Line 157: What do the authors mean by “mixed method survey” Likert and open-ended questions? This is unclear. What are the participant groups?...referrers and staff? | This section has been reworded to clarify that each survey included Likert items and space for open comments. Additional information has been added in other areas of the manuscript to provide further detail regarding the participant groups. |
| Results This section needs to be made much more concise. Also, it is difficult to understand the tables without referring to the supplemental document. The tables could be made clearer with the additional explanation. For example, Table 3 “complexity-duration” is this duration of the ESD program? What aspect? “Complexity-degree of difference”—the reader has no context for this. What does this mean? | A brief description of each domain and construct has now been added into the Methods section to provide easier access to these terms for readers. |
| Consider making the tables clearer and then incorporating a few of the quotes into the tables. This would significantly cut down on the word count in this section. The tables seem to tell most of the story, the pages upon pages of words are extraneous. | The results section has now been condensed, and quantitative / qualitative findings amalgamated into the tables. However, additional qualitative data remains in this section to add further context and details to the quantitative findings. We disagree that the tables in themselves tell most of the story. As a mixed methods study, the quantitative and qualitative data is intended to be complimentary and of equal importance. |
| At the end of the section the authors highlight ‘key constructs’. It is unclear how these were derived. However, the authors might consider explaining how they came up with these key concepts and then focus on these areas (using words) in the results section. Let the tables tell the story of the data for all the other areas. | Further details have been added to the methodology to explain how the key relationships between constructs were derived. As stated above, we believe the tables alone are insufficient to present the findings for the domains, and have elected to retain some of the the additional information in these sections. |
| Discussion Line 612: What is the champion? | Additional information has been added to the manuscript to clarify this title. |
| There is an entire paragraph written about the perception of patients ‘missing out’—what does this mean? Again, there is no context for the reader to understand this notion. | This paragraph has now been removed |
| Consider linking the topics in the discussion with some transition sentences to improve the flow. This section reads like an extension of the results section. | Additional linking sentence has been added to improve the flow of this section. |
| Consider removing the paragraph lines 657-680. There is no need to continue a defence of the study methodology (use of CFIR) here. This was all covered earlier in the paper (although it needs to be clarified). | This paragraph has now been removed |
