# Supplementary material for: Evaluating the implementation of an early supported discharge (ESD) program for stroke survivors: A mixed methods longitudinal case study
Source: PLoS One. 2020 Jun 24;15(6):e0235055. doi: 10.1371/journal.pone.0235055 (PMC7313954; doi:10.1371/journal.pone.0235055)
Supplement: Attachment — Submitted filename: Response to Reviewers 1.docx [file pone.0235055.s005.docx]

Dear Academic Editor Tan,

**RE:	PONE-D-19-26093R1 - Evaluating the implementation of an Early Supported Discharge (ESD) program for stroke survivors: A mixed methods longitudinal case study**

Thank you for your email dated March 3rd, 2020 regarding this manuscript. We have endeavoured to answer the remaining reviewer’s queries and recommendations to the best of our ability, and please find attached a point-by-point response to all of their comments.

We look forward to continuing to participate in the peer review process with PLOS One in the coming weeks.

Kind regards,

Dr. Danielle Hitch

Corresponding Author

| **Reviewer 1** |  |
| --- | --- |
| The authors state that they feel that they are unable to substantially reduce the word count because they would lose "essential information". Yet the excessive text actually takes away from this paper. The reader is completely bogged down in the minutia and all the quotes to the point where one simply loses interest. The results of this study simply do not warrant over 7,000 words of text. | A further edit has been undertaken throughout this manuscript and the overall word count (including all aspects of the document) is now 7737. This represents a further 16% reduction on the previous version, and we genuinely don’t believe we can remove any more content without significantly impacting on the presentation of this study. |
| The intro can be synthesized, the reader does not need to read a long paragraph describing previous work study by study. | Sections of the introduction have been further synthesised to present his background information more succinctly. |
| This reviewer asked for the authors to include a description of their specific ESD program. The reader still does not know how the referral process works, who delivered that care in the home, for how long was care provided, was the care daily or weekly, how were patients selected for ESD, what the discharge process from ESD, was there continuing care after discharge from ESD? Without an in depth understanding of the program, this data is meaningless. The reader doesn't know anything about the service that the staff is being surveyed about. It truly renders these results meaningless. | We have now added into additional information that specifically addresses the questions you have posed in your response. |
| The reviewer, once again, suggests using the table to in the results section and highlighting the significant findings with text.  *We returned to your previous suggestion, as the wording of your recommendation was unclear.*  Previously you recommended:  Consider making the tables clearer and then incorporating a few of the quotes into the tables. This would significantly cut down on the word count in this section. The tables seem to tell most of the story, the pages upon pages of words are extraneous.  At the end of the section the authors highlight ‘key constructs’. It is unclear how these were derived. However, the authors might consider explaining how they came up with these key concepts and then focus on these areas (using words) in the results section. Let the tables tell the story of the data for all the other areas.” | In our previous revision, we had:   - Provided a brief description of each domain and construct in the Methods section to provide easier access to those terms for readers - Condensed the results section and amalgamated quantitative and qualitative findings into tables. - Added additional information in regards to how the key constructs were derived.   We assumed that you disagree with our preference to retain quotes in the body of the article, and have therefore removed the majority of them. The only exceptions have been brief quotes within sentences and the Outer Setting section (which did not have a table). All elaborative prose beneath each table has also been condensed. |
